# Adherence to the nurse-driven hemodynamic protocol during postoperative care

**DOI:** 10.1186/cc13328

**Published:** 2014-03-17

**Authors:** J Klimes, J Hruda, M Lukes, P Suk, V Sramek

**Affiliations:** 1 St Anna University Hospital and International Clinical Research Center, Brno, Czech Republic

## Introduction

The aim of this work is to verify that low incidence of hemodynamic interventions in hemodynamically monitored patients in our postoperative ICU is not caused by insufficient attention being paid to the drop of noncalibrated cardiac index (nCI). In the last 2 years there was a need for hemodynamic intervention in 92% of patients with perioperative cardiac output monitoring, while only 31% of these patients needed at least one hemodynamic intervention postoperatively in the ICU.

## Methods

High-risk patients planned to undergo elective major abdominal surgery were routinely monitored with LiDCOrapid; the monitoring continues overnight after surgery. The target nCI for the postoperative monitoring, as well as hemodynamic interventions in case of its drop, were prescribed by the anesthetist in the protocol upon handover of the patient to the postoperative ICU. The hemodynamic protocol in the ICU was then nurse driven with compulsory recording of patient's hemodynamic data into the patient's documentation every 2 hours and at the time of each intervention. Data from the memory of the LiDCOrapid monitor were analyzed using LiDCOview software and compared with patients' documentation.

## Results

A total 649 hours of hemodynamic record were analyzed in 40 patients chosen at random from 121 patients monitored in the last 2 years. We found 22 drops in nCI that were noticed by the nursing staff and led to volume challenge in accordance with the protocol. One-half of these interventions was carried out outside the 2-hour interval of protocol-required monitoring and documentation entry. We identified nine drops in nCI that were not reacted to. None of them happened within the first 4 hours postoperatively and all of them were outside the 2-hour interval of protocol-required monitoring. In five of these episodes the pulse pressure also dropped, suggesting impairment of the reading of the waveform from the arterial line.

## Conclusion

The need for hemodynamic intervention in postoperative care was quite rare in our patients. The drop in nCI was noticed in most of the cases and the patient was treated according to the protocol. However, isolated episodes of nCI drop were missed during the night after the operation. The results were pointed out to the nursing staff and a comparative survey will be conducted. As with any monitoring modality, (non)adherence to the protocol may become the limiting issue to the benefit for the patient.

